# Supplementary material for: Mixed and nonvaccine high risk HPV types are associated with higher mortality in Black women with cervical cancer
Source: Sci Rep. 2021 Jul 7;11:14064. doi: 10.1038/s41598-021-93485-1 (PMC8263581; doi:10.1038/s41598-021-93485-1)
Supplement: Supplementary file 1 — Supplementary Figures. [file 41598_2021_93485_MOESM1_ESM.pdf]

Supplementary Information

Mixed and nonvaccine high risk HPV types are associated with higher mortality in Black women with cervical cancer

Rachelle P. Mendoza[1], Tahmineh Haidary[1], Elmer Gabutan[3], Yin Ying Zhou[2], Zaheer Bukhari[1], Courtney Connelly[1], Wen-Ching Lee[2], Yi-Chun Lee[2], Raj Wadgaonkar[3], Raag Agrawal[1], M.A. Haseeb[1,3], Raavi Gupta[1]✉

[1]Department of Pathology
[2] Department of Obstetrics and Gynecology
[3] Department of Medicine
State University of New York, State University of New York, Downstate Health Sciences University, Brooklyn, NY 11203, U.S.A.

Supplementary Figure (SF 1)

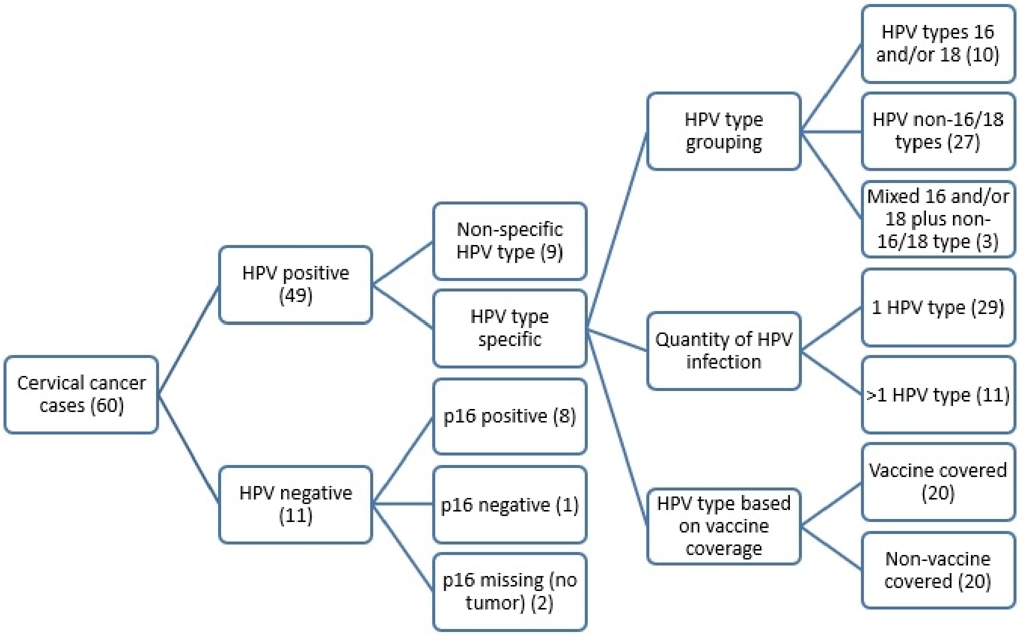

Supplementary Figure 1 (SF 1). Schematic representation of patient groups relative to HPV genotypes. **[Microsoft Excel 2019 (version 1808)].**
