# Supplementary material for: Mixed and nonvaccine high risk HPV types are associated with higher mortality in Black women with cervical cancer
Source: Sci Rep. 2021 Jul 7;11:14064. doi: 10.1038/s41598-021-93485-1 (PMC8263581; doi:10.1038/s41598-021-93485-1)
Supplement: Supplementary file 2 — Supplementary Tables. [file 41598_2021_93485_MOESM2_ESM.pdf]

Supplementary Information

Mixed and nonvaccine high risk HPV types are associated with higher mortality in Black women with cervical cancer

Rachelle P. Mendoza[1], Tahmineh Haidary[1], Elmer Gabutan[3], Yin Ying Zhou[2], Zaheer Bukhari[1], Courtney Connelly[1], Wen-Ching Lee[2], Yi-Chun Lee[2], Raj Wadgaonkar[3], Raag Agrawal[1], M.A. Haseeb[1,3], Raavi Gupta[1]✉

[1]Department of Pathology
[2] Department of Obstetrics and Gynecology
[3] Department of Medicine
State University of New York, State University of New York, Downstate Health Sciences University, Brooklyn, NY 11203, U.S.A.

Supplementary Table (ST 1). Primers used for the PCR analysis of high risk HPV genotypes*.

Name	Forward primer sequence	Reverse primer sequence	size (bp)
16-1	TTAGGCAGCACTTGGCCAACCA	TAATCCGTCCTTTGTGTGAGCT	207
16-2	ACTGCAATGTTTCAGGACCCAC	CGAAGCGTAGAGTCACACTTGC	661
18-1	TCGCGTCCTTTATCACAGGGCGA	TGCCCAGGTACAGGAGACTGTG	536
18-2	TCCGTGGTGTGCATCCCAGCAG	CACTTGTGCATCATTGTGGACC	274
26-1	TGGTATACAACGAGTGTCAGCTCC	GGGGCAATGATGGCCATGTCG	635
31-1	AGGCACGGTTGGTGAATCGGTC	TAGATGCTGAGGGTGCACTACG	683
31-2	CATGAACTAAGCTCGGCATTGG	TCCAACATGCTATGCAACGTCC	385
33-1	AGCTTAGAGGTGTGGCTTTGTG	TGCAGTTAGTTGCAGTACGTGC	493
33-2	TGACCCACCTACAGCTGCAATC	GGGTGTGTACATTATCCACATCG	899
35-1	CCACCAAGTGGTTCCAACGCAG	TGTAGGCGTGTAGCTGTGTAGC	488
35-2	GTCCTGTTGGAAACCAACACGT	ACACACAGACGTAGTGTCGCCT	251
39-1	ACACAAACGGTGTATTCCGTGCCA	TGTGCAGTTGGAGATTTGGGATCC	200
39-2	TGTGCAGTACCAGTGACGGATCG	ATTTTTGGCGTTGTGACTCTGTG	438
45-1	GGACATCACACCTACCGTGGAC	CTGTGAGGTGGACACACGGACC	298
45-2	ACCTGCACAATTGCAACCTGGT	CAACTGCCAGGGGTTTCACGCA	345
51-1	AATTGCTGGCAACGTACACGAC	ACACTTGAACACCTGCAACACG	255
51-2	CCTACTCCAGGGGTTAGTCGCA	TAAGGAGGGCAACTGCCTAGAC	504
52-1	CCCAAGTGTAACGTCATGCGTG	AGGGTTGTTTATAGCCGTGCAC	323
52-2	ACCTCCGCAGTGTCCGTGGGTG	AAGAGCGGCCTAAGCACTGCAC	601
53-1	TTGTTCAGTGTACGGGGCTAGC	GTGACGCCATTGCAGTTATCGCCT	549
53-2	TTCTGCAGTAAGCTATGAGGGCAT	AACCACTGTCGATTTCGGTGTT	449
56-1	CTGGGCACTAGGTCAAAGCCTGCT	CAACCACGCGTAAAAGCACTCAT	307
58-1	GGTAGTACCCCACCGTCTGAGG	AGACGTGACATTGCCACTGTCA	414
58-2	ACCAGACTCCAGAGACAACACC	TCACCTTTGTCATCACTGGTCC	264
59-1	AGACACCGTTACATGAGCTGCT	TCATTCTCGGAGTCGGAGTCAG	320
59-2	TCTAACGCCATCTGCAGCAAGG	ACAGTAGTCCACTGACACGCTG	438
66-1	TGCGGTAGTATCCTTGGGCAGTG	TACAATAAGGGCTACACGCCAA	388
68-1	GGTACTGCTTGGAACACGCCTG	GGCCCCCAGACATAGGGACCTT	368
68-2	GTCAAAAAGACGCCCCTGCACCTA	CACACCTTAGGGTAGGGCTACAA	490
73-1	ACAGGCTATTAGTTGCCAACGTC	TTCTTAGGTGTGGCACTTGTG	222
73-2	GGGGTGGGCAAAGGTAGGTAGC	ACAATCCAGGGGCCTCTGGTCCGA	322
82-1	TGTCCGTGGACACCTGCGACCA	GTAGTTAAAGGTGATGTGGCAACC	546
82-2	CCCAAAACCAATACACGTGCTGAA	AACATCCTGTTGGTCGTTGCCA	270
β-globin	GAAGAGCCAAGGACAGGTAC	CAACTTCATCCACGTTCACC	268

* Muñoz N. *et al.* Epidemiologic classification of human papillomavirus types associated with cervical cancer. *N. Engl. J. Med.* **348**, 518–527. doi:10.1056/NEJMoa021641. PMID 12571259 (2003). Of the 120 known human papilloma viruses, 51 species and three subtypes infect the genital mucosa. 15 are classified as high-risk types (16, 18, 31, 33, 35, 39, 45, 51, 52, 56, 58, 59, 68, 73, and 82), three as probable high-risk (26, 53, and 66), and 12 as low-risk (6, 11, 40, 42, 43, 44, 54, 61, 70, 72, 81, and CP6108).
